# Associations between plasma proteomic signatures and secondary sleep in older adults

**DOI:** 10.1007/s11357-025-01565-1

**Published:** 2025-04-08

**Authors:** Kaushalya Madhawa, Thomas Svensson, Hoang Nt, Ung-il Chung, Akiko Kishi Svensson

**Affiliations:** 1 Precision Health, Department of Bioengineering, Graduate School of Engineering, The University of Tokyo, 7-3-1 Hongo, Bunkyo-ku, Tokyo 113-8656 Japan; 2 Graduate School of Health Innovation, Kanagawa University of Human Services, Kawasaki-ku, Kawasaki-shi, Kanagawa 210-0821 Japan; 3 Department of Clinical Sciences, Lund University, Skåne University Hospital, Malmö, Sweden; 4 Clinical Biotechnology, Center for Disease Biology and Integrative Medicine, Graduate School of Medicine, The University of Tokyo, Tokyo, Japan; 5 Department of Diabetes and Metabolic Diseases, The University of Tokyo, 7-3-1 Hongo, Bunkyo-ku, Tokyo 113-0033 Japan

**Keywords:** Sleep, Proteomics, Biomarkers

## Abstract

Sleep disturbances are prevalent among elderly populations and are linked to various health complications. Understanding the underlying biological mechanisms contributing to sleep disorders is crucial for developing targeted interventions. In this study, we measured 355 plasma proteins in an elderly Japanese cohort (*n*=77) using a high-throughput proteomic platform. Additionally, we collected over 25,000 person-days of physical activity and sleep behavior data from wrist-worn wearable devices, focusing on total sleep time (TST) across 24 h and daytime sleep. Fragmented sleep was observed as one of the most prevalent sleep disturbances in this population. In protein expression analysis, we identified 9 protein biomarkers associated with increased secondary sleep TST, defined as additional sleep episodes outside of the main sleep episode within 24 h. These findings may suggest disruptions in circadian rhythms or underlying health conditions. Functional analysis revealed that biological processes related to inflammation play a significant role in regulating sleep behavior. Further analysis showed an association of 12 proteins with daytime sleep and 5 proteins with afternoon sleep. Overall, this study identified inflammatory biomarkers and biological processes associated with sleep behavior in the elderly, presenting promising opportunities for developing diagnostic tools and targeted clinical interventions.

**Supplementary Information:**

The online version contains supplementary material available at 10.1007/s11357-025-01565-1.

## Introduction

Sleep is crucial for maintaining overall health, particularly in the elderly, whose sleep quality significantly influences their well-being. Disruptions of sleep patterns have been linked to various adverse health outcomes including cognitive decline, anxiety [1], type-2 diabetes [2], cardiovascular diseases [3], and obesity [4]. Some common sleep disorders experienced by elderly adults include sleep deprivation, fragmentation of sleep by periods of wakefulness, and increased nighttime wakefulness [5]. Sleep disorders experienced by the elderly can be attributed to normal aging processes, co-morbid medical conditions, genetics [6], and the use of certain medications [7, 8]. A person may experience multiple sleep episodes during the day due to several factors such as sleep fragmentation and daytime napping which can be interrelated [9]. Sleep fragmentation [10] involves frequent interruptions during the main sleep period at night, leading to diminished sleep continuity and quality. Fragmented sleep has significant health implications, including increased risks of metabolic disorders [11, 12], cardiovascular diseases [13], and impaired cognitive function [14]. Previous research suggests that sleep fragmentation can be considered an early symptom of Alzheimer’s disease and Parkinson’s disease [15].

Meanwhile, multiple sleep episodes including daytime naps can occur as a compensatory response to inadequate or poor-quality nighttime sleep. However, daytime napping may also reflect cultural or personal habits that are not necessarily driven by nighttime sleep disturbances. While daytime napping is often studied, it represents only one aspect of daily sleep behavior and relying solely on self-reported questionnaires can hinder our ability to capture the full range of discrete sleep episodes throughout the day. Our goal is to understand these additional sleep episodes—whether they take the form of short naps or longer bouts of secondary sleep—occurring outside the main sleep period. Wearable devices offer a more comprehensive approach by accurately tracking unplanned sleep episodes that may otherwise go unnoticed by self-report methods, offering valuable insights into their sleep patterns.

Despite its importance, the causal factors underlying sleep-related issues in the elderly remain poorly understood. Understanding biological mechanisms responsible for different sleep phenotypes is crucial in discovering potential biomarkers and designing interventions for improving sleep quality. Several genome-wide association studies (GWAS) have discovered genetic variants associated with sleep duration [16], insomnia [17, 18], and sleep chronotype [19, 20]. While GWAS can identify the genetic variants associated with a trait, they do not directly reveal how these variants influence protein expression and interactions. In contrast, proteomics provides a complementary perspective by measuring protein abundance levels. Recent developments in high-throughput proteomic platforms [21, 22] have enabled simultaneous measurement of thousands of proteins from a single sample. Changes in the expression of certain proteins can influence sleep patterns [23, 24] which may in turn adversely affect overall health. Moreover, identifying proteomic biomarkers related to sleep behavior could improve our understanding of how sleep quality and disorders are associated with aging-related biological processes and health conditions.

However, most studies on physical activity and sleep patterns rely on self-reported data, which is prone to low reliability, particularly among the elderly, where discrepancies between perceived and actual behaviors can be significant [25, 26]. It is important to capture the sleep behavior of participants during the day, especially if they are having sleep disturbances. Although polysomnography (PSG) is considered the gold standard in measuring sleep, the number of nights monitored is limited due to time and monetary constraints [27]. Actigraphy devices are used as an alternative for measuring sleep. However, their use is limited to 7 to 14 [16, 28] days which may not accurately reflect long-term behaviors. On the other hand, commercially available wearable devices offer a convenient alternative by automatically logging physical activity and sleep data as they can be worn continuously throughout the day. Validation studies indicate that modern multi-sensor wearable devices are comparable or superior to traditional actigraphy [29] in measuring sleep variables. The current work aims to identify plasma proteins and pathways associated with different sleep patterns in elderly adults.

## Methods

### Study design

#### Study participants

One hundred fifty generally healthy older adults were eligible for inclusion in the cohort, all of whom were over 80 years of age (mean age (± (SD)) = $85.8 (\pm 4.4)$ years), free from clinically significant illnesses, and requiring care-need level 1 or lower as classified by the Japanese care level system [30]. All participants in this study were community-dwelling individuals, and all testing and data collection were conducted at an outpatient clinic in a hospital setting. The baseline assessment including plasma sample collection was conducted from 10th October 2022 to 26th December 2022. The participants were recruited through an advertisement posted on multiple Japanese newspapers. They were asked to fast more than six hours before the visit. Additionally, details about their last meal were recorded. Participants were instructed to use wearable devices continuously and to complete daily and monthly lifestyle questionnaires while in free-living conditions. Participants’ demographic information and existing medical conditions were obtained using a baseline questionnaire at the beginning of the study. Activity levels and sleep behaviors were continuously tracked using a Fitbit Inspire 2^1^ wrist-worn fitness tracker. Participants were assessed for cognitive impairment using the Japanese version of the Montreal Cognitive Assessment (MoCA-J) score [31, 32]. Their lower extremity strength and functional mobility were measured using the five-repetition sit-to-stand (SS-5) score [33]. A random sample of 88 participants was selected from this cohort for measuring plasma protein abundance levels. Eighty-eight samples is the maximum number of samples accommodated by a single Olink Panel kit [21]. We excluded one person diagnosed with chronic kidney disease.

#### Proteomic markers

**Fig. 1:**
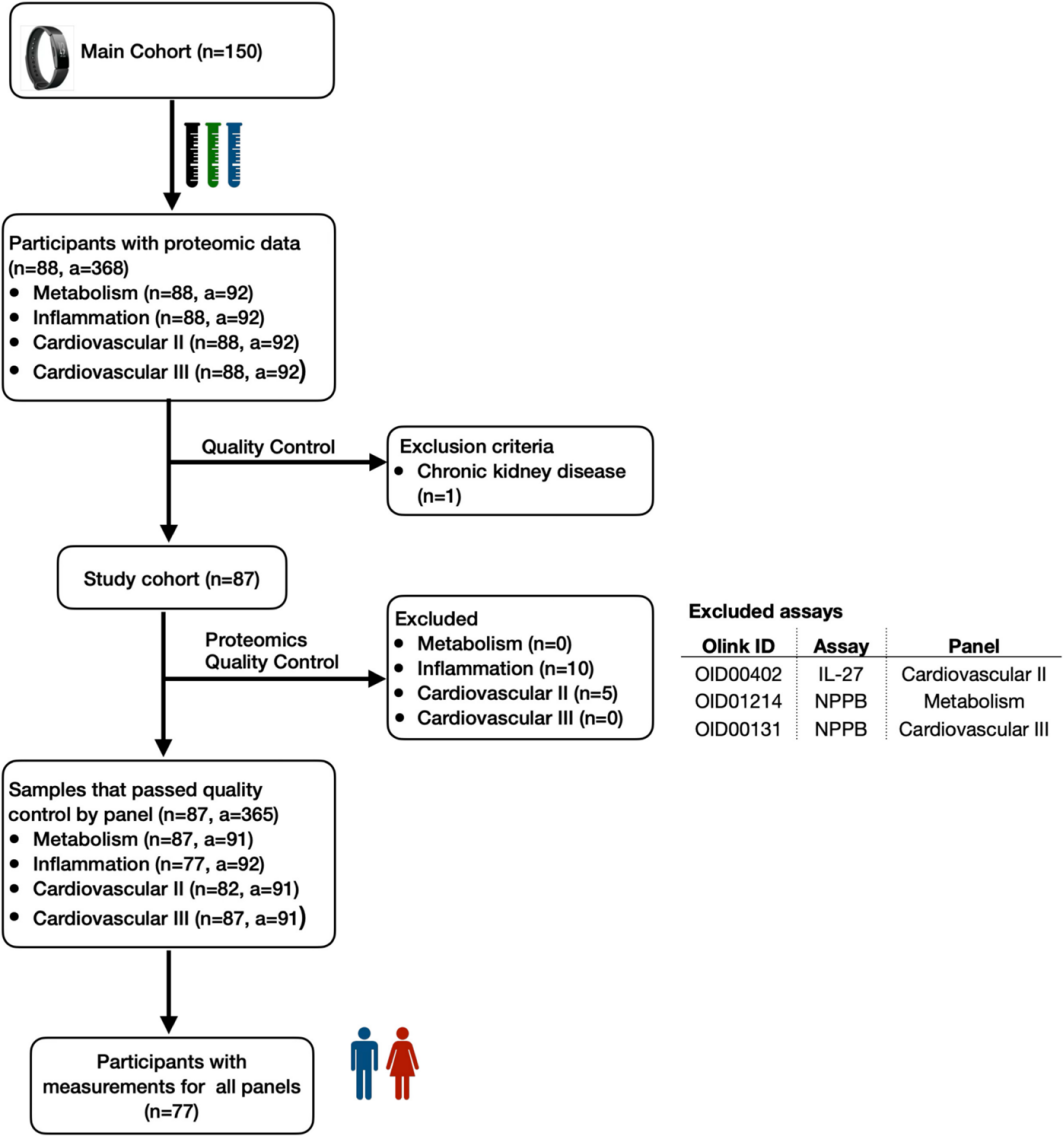
**Flowchart of participant inclusion and exclusion**. A sample of 77 participants was selected from an elderly Japanese cohort of 150 participants. We measured the abundance levels of 368 plasma proteins using proximity extension assay (PEA) technology. After excluding samples that failed quality control, we analyzed 365 assays, with sample counts varying across these assays (*n*, number of participants; *a*, number of assays)

EDTA-treated plasma samples were collected to quantify plasma protein abundance levels using four Olink Target 96 panels^2^: Cardiovascular II, Cardiovascular III, Inflammation, and Metabolism, each consisting of 92 assays, totalling 368 assays. The plasma samples were frozen to $-80~^\circ \text {C}$ immediately after collection and shipped to Olink Analysis Service in Sweden for analysis. The abundance levels of plasma proteins were quantified using the Proximity Extension Assay (PEA). Four internal controls were added to each sample to ensure inter- and intra-plate quality control (QC). We excluded measurements with QC warnings, resulting in 365 assays for analysis, with sample counts varying across assays. It is important to note that two assays from different panels can measure the same protein; thus, these 365 assays measure the abundance levels of 355 distinct proteins. The number of participants remaining after QC filtering is detailed in Fig. 1. Ultimately, 77 participants with valid measurements across all four panels were included in the analysis. All 77 participants were 80 years or older, and 31 were 90 years or older at the time of data collection. Protein abundance levels were measured in normalized protein expression (NPX) [21] units on a $\text {log}_2$ scale.

#### Wearable device

We used Fitbit wearable data collected from 28th October 2022 to 26th December 2023 accounting for 425 days. On average, a single participant was observed for 365.3 ($\pm 32.3$) days. The Fitbit wearable device logs sleep episodes that last at least an hour. To ensure data quality, we excluded days where a participant’s 24-h total sleep time was recorded as less than 2 h. Additionally, for the present study, we required each participant to have at least 90 days of sleep and activity data available to be included in the analysis. The longest sleep episode within 24 h is designated as the main sleep. While nighttime sleep is typically classified as the main sleep, it can occur during the day if the participant sleeps longer in the daytime.

Fitbit devices utilize two distinct sleep detection algorithms: the classic sleep algorithm and the newer sleep stages algorithm [34]. The classic algorithm relies primarily on movement data detected through an accelerometer to classify sleep periods. While effective for basic sleep tracking, this algorithm can inaccurately label periods of inactivity as sleep due to its sole dependence on movement. In contrast, the sleep stages algorithm combines movement data with heart rate variability (HRV) measured through optical heart rate sensors. This allows the Fitbit device to classify sleep into three sleep stages—deep, rapid eye movement (REM), and light, based on movement and heart rate patterns [34]. The Fitbit sleep stage light corresponds to N1+N2 sleep and deep corresponds to N3 sleep [35].

In this study, we chose to use only the sleep stages data due to the higher accuracy as it is essential to ensure that our analysis reflected true sleep behavior, minimizing the risk of mislabeling inactive periods as sleep and enhancing the validity of our findings.

We obtained sleep records of each participant for the entire study period. We calculated the total sleep time (TST) for each sleep episode where a sleep episode is defined as any continuous period during which the participant was asleep. TST considers only the time spent in sleep stages excluding any awake periods within a sleep episode. It has been shown that the TST measured by Fitbit devices with sleep staging does not show a significant difference compared to PSG [36]. By summing the TSTs of all sleep episodes within 24 h, we obtained the 24-h TST. For each participant, we then calculated the daily average 24-h TST by dividing the cumulative 24-h TST over the entire period by the number of days recorded by the wearable device.

**Fig. 2:**
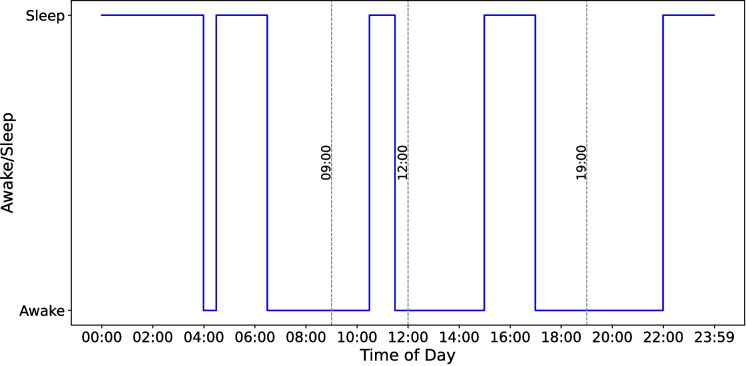
**Illustration of main, secondary, daytime, and afternoon sleep classification.** Sleep logs belonging to a hypothetical participant, illustrating the classification of main sleep, secondary sleep, daytime sleep, and afternoon sleep. The participant has four sleep episodes within 24 h. The main sleep episode is the longest continuous sleep period, occurring from 22:00 to 4:00 the next day. The three remaining sleep episodes are classified as secondary sleep. Among these, two sleep episodes between 9:00 and 19:00 are considered daytime sleep, with the sleep episode from 15:00 to 17:00 further classified as afternoon sleep

We categorized sleep episodes occurring outside the main sleep episode as secondary sleep. We divided secondary sleep episodes into daytime and nighttime based on sleep onset and wake-up times. We defined sleep episodes starting on or after 09:00 and ending before 19:00 as daytime sleep. We further classified daytime sleep episodes starting on or after noon as afternoon secondary sleep and the sleep episodes starting on or after 9:00 and ending before noon as morning secondary sleep. For each participant, in addition to the average 24-h TST, we separately computed the average TSTs for main sleep, secondary sleep (secondary sleep TST), daytime secondary sleep (daytime TST), and morning secondary and afternoon secondary sleep (afternoon TST). We standardized each continuous variable across all participants by subtracting its overall mean and dividing by its standard deviation, thereby transforming the data to have a mean of zero and a standard deviation of one.

In Fig. 2, we show four sleep records logged by a hypothetical participant within 24 h. According to this figure, the main sleep episode is 6 h from 22:00 to 04:00. The remaining three sleep episodes (04:30–06:30, 10:30–11:30, and 15:00–17:00) contribute to 5 h of secondary sleep TST. Two of the secondary sleep episodes (10:30–11:30 and 15:00–17:00) contribute to 3 h of daytime TST. The sleep episode starting at 10:30 and ending at 11:30 will be classified as morning sleep while the sleep episode starting at 15:00 and ending at 17:00 is classified as afternoon sleep, contributing 1 h of morning sleep and 2 h of afternoon TST.

**Fig. 3:**
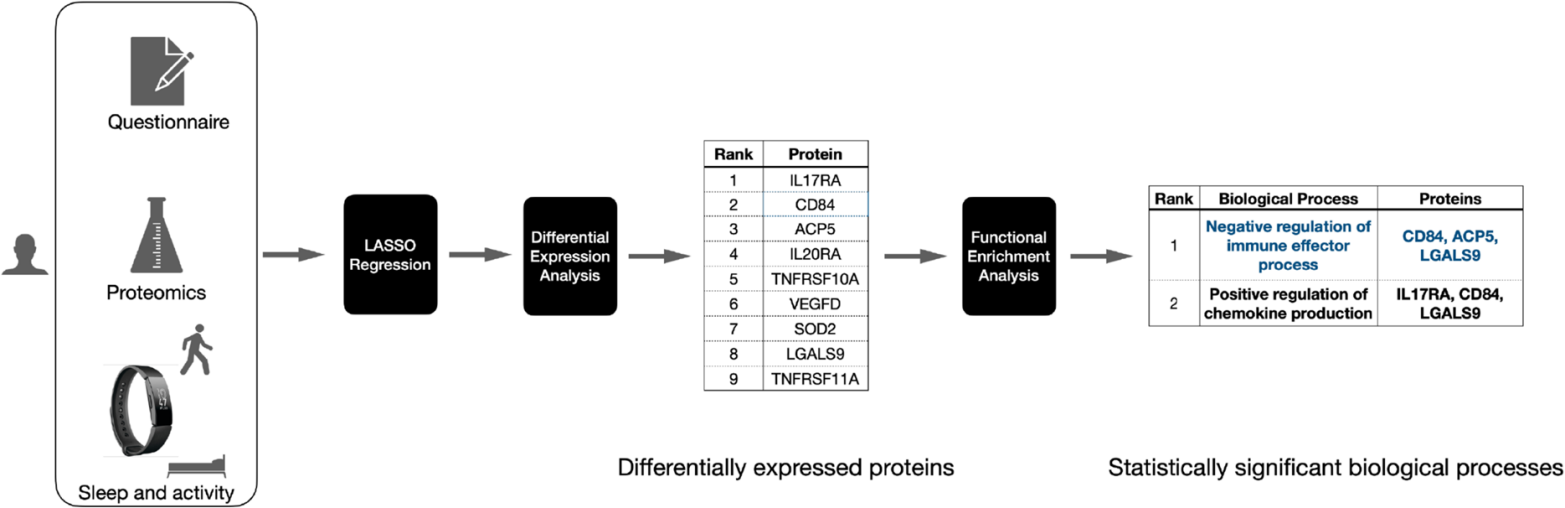
**The biomarker and pathway discovery workflow.** We performed differential expression analysis using protein expression levels measured using the Olink Target 96 panels alongside sleep data obtained from Fitbit wearable devices to identify protein biomarkers associated with secondary sleep TST (in minutes). Statistically significant proteins were selected based on an adjusted *p*-value of $< 0.05$. These significant proteins were further analyzed using functional enrichment analysis with Metascape to uncover the biological pathways they are associated with

### Statistical analysis

First, we investigated the role of protein biomarkers related to sleep habits. The details of this analysis workflow are depicted in Fig. 3. We employed a two-step approach to identify significant protein biomarkers. First, we fitted LASSO regression [37] to predict the secondary sleep TST using protein abundance levels and demographics variables. We shortlisted a subset of proteins by selecting those with non-zero coefficients, effectively reducing the number of proteins considered. The assays with non-zero coefficients were then independently analyzed using linear regression models to assess their significance. This analysis is performed using the limma package [38] in R, focusing on the association between the normalized protein expression levels (NPX) and the secondary sleep TST for each participant. We fitted a linear regression model for each protein assay to assess the association between the normalized NPX value of the protein and the secondary sleep TST. The model was adjusted for confounding variables including age, sex, body mass index (BMI), average daily step count, and specific medical conditions such as metabolic diseases, hypertension, and arrhythmia/heart failure, which were treated as binary covariates. Additionally, blood Cystatin C levels were included as a covariate to serve as a marker of kidney function. A complete list of these covariates is provided in Table 1. To account for multiple comparisons, *p*-values were adjusted using the Benjamini-Hochberg false discovery rate (FDR) method [39], with an adjusted *p*-value (*q*) threshold of 0.05 for significance. Variance estimates were stabilized using the empirical Bayes method [40]. We independently performed this LASSO regression followed by differential expression analyses to further understand the relationship between protein expression levels and the timing of sleep episodes, focusing on daytime and afternoon sleep episodes. All models were adjusted for the same set of variables as mentioned previously. As an additional analysis, we also considered potential nonlinear relationships between secondary sleep TST and protein expression levels by incorporating quadratic terms of secondary sleep TST into the model.

**Table 1:** Participant characteristics. Values are means (SD) for continuous variables and counts (%) for categorical variables

Variable	Summary
*n*	77
Age	87.6 (4.2)
Female, *n* (%)	45 (58.4)
BMI	22.6 (3.5)
Average daily step count	4968.5 (2846.7)
Grip strength dominant hand (kg)	22.7 (7.9)
MoCA-J score	21.8 (3.7)
SS-5 score (seconds)	11.5 (3.3)
Cystatin C (mg/L)	1.2 (0.3)
Disease under treatment, *n* (%)	
Hypertension	29 (37.7)
Metabolic diseases	8 (10.4)
Arrhythmia/heart failure	3 (3.9)
Ischemic heart disease	6 (7.8)
Nervous system	1 (1.3)
Digestive system diseases	5 (6.8)
Renal and urinary system	5 (6.5)
Psychological conditions	1 (1.3)
Allergy	2 (2.6)
Need nursing care	
Not required	60 (77.9)
Support required - level 1	8 (10.4)
Support required - Level 2	8 (10.4)
Long-term care level 1	1 (1.3)
Average sleep time (minutes)	
Main sleep	374.04 (54.2)
Secondary sleep	6.1 (7.8)
Total sleep	380.14 (54.9)

Next, we investigated the biological processes associated with the differentially expressed proteins. We performed functional enrichment analysis on the set of significant assays using Metascape [41] with gene ontology (GO) [42, 43] biological processes (BP) and the Reactome pathway knowledgebase [44] as ontology sources. The set of genes corresponding to the 355 distinct protein assays was used as the background gene set. In this analysis, we used the default parameters to select terms with a *p*-value $< 0.01$, a minimum count of 3, and an enrichment factor $> 1.5$. These terms were grouped into clusters based on their membership similarities. We used Kappa scores as the similarity metric to perform hierarchical clustering on the enriched terms such that subtrees with a similarity of $> 0.3$ are considered distinct clusters. The most statistically significant term in a cluster was selected to represent the cluster.

## Results

The average age of the participants was 87.6 ($\pm 4.2$) years. Their average body mass index (BMI) was 22.6 ($\pm 3.5$) $kg/m^2$, and their average grip strength was 22.7 ($\pm 7.9$) kg. The average MoCA-J score was 21.8 ($\pm 3.7$) points. Complete participant characteristics are provided in Table 1.

Of the 77 participants, 67 had at least a single secondary sleep episode recorded in addition to their main sleep episode. Additionally, 35 participants had at least one daytime sleep episode (09:00–19:00), and 30 had at least a single afternoon sleep episode (12:00–19:00). On the other hand, we did not observe any participant having a sleep episode starting on or after 9:00 and ending on or before noon the same day.

**Fig. 4:**
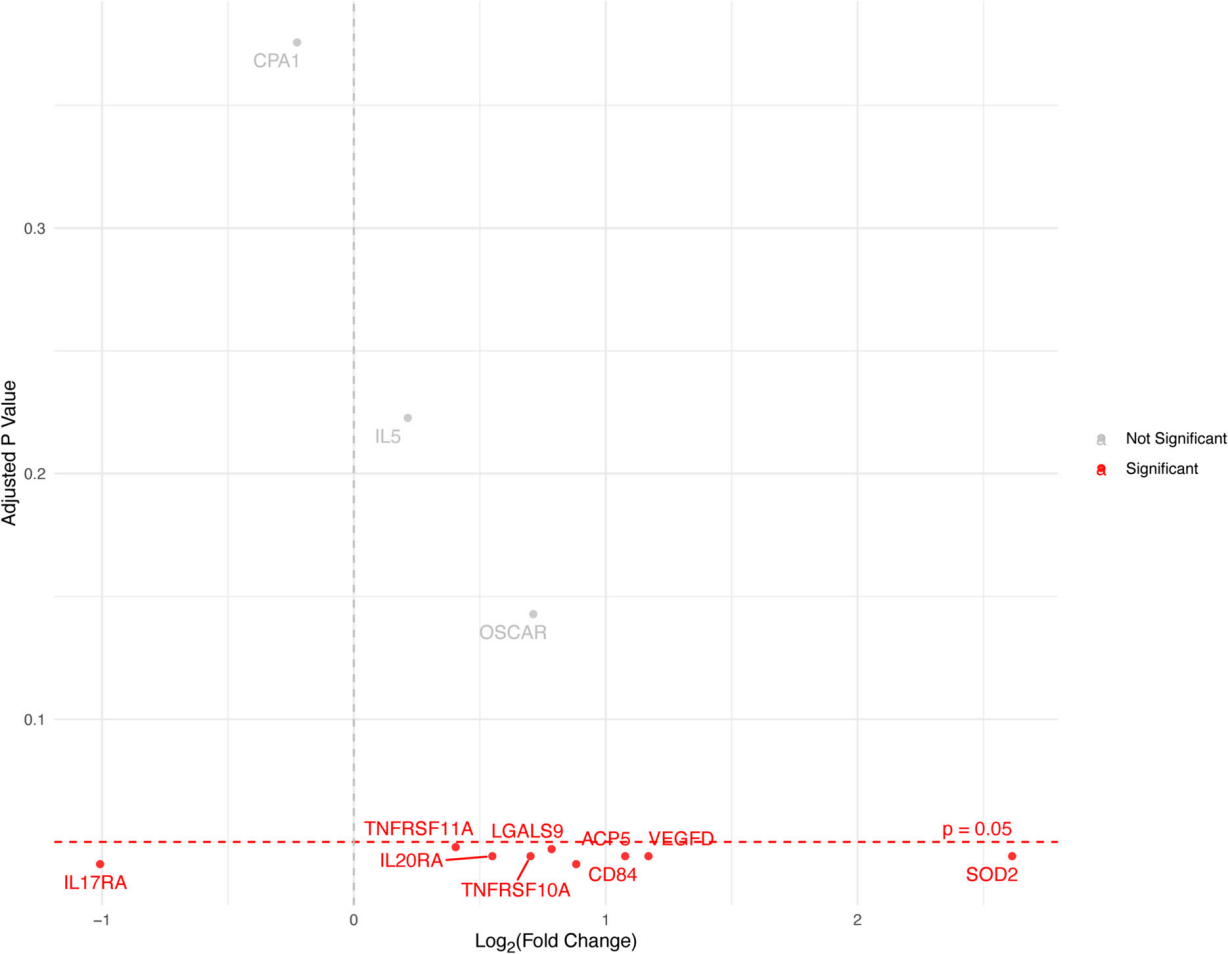
**Differential expression analysis of plasma proteins with secondary sleep TST of participants.** Proteins significantly dysregulated with a *p*-value of 0.05 after adjusting for multiple-testing with Benjamini-Hochberg false discovery rate (FDR) are colored in red

The LASSO regression resulted in 15 proteins with non-zero coefficients for the secondary sleep TST. With differential expression analysis, we identified that 9 out of these 15 protein assays exhibited significant dysregulation ($q < 0.05$) in relation to the secondary sleep TST. The volcano plot in Fig. 4 shows all 9 proteins with a statistically significant dysregulation. As shown in the figure, eight proteins were up-regulated while Interleukin-17A (IL17A) was the only protein down-regulated with secondary sleep TST. The analysis considering the quadratic terms of secondary sleep TST did not reveal any significant nonlinear relationships with protein expression levels.

The functional analysis revealed two biological processes to be statistically significant ($p < 0.01$): negative regulation of the immune effector process and the positive regulation of chemokine production. The statistically significant biological processes and proteins belonging to each pathway are shown in Table 2.

**Fig. 5:**
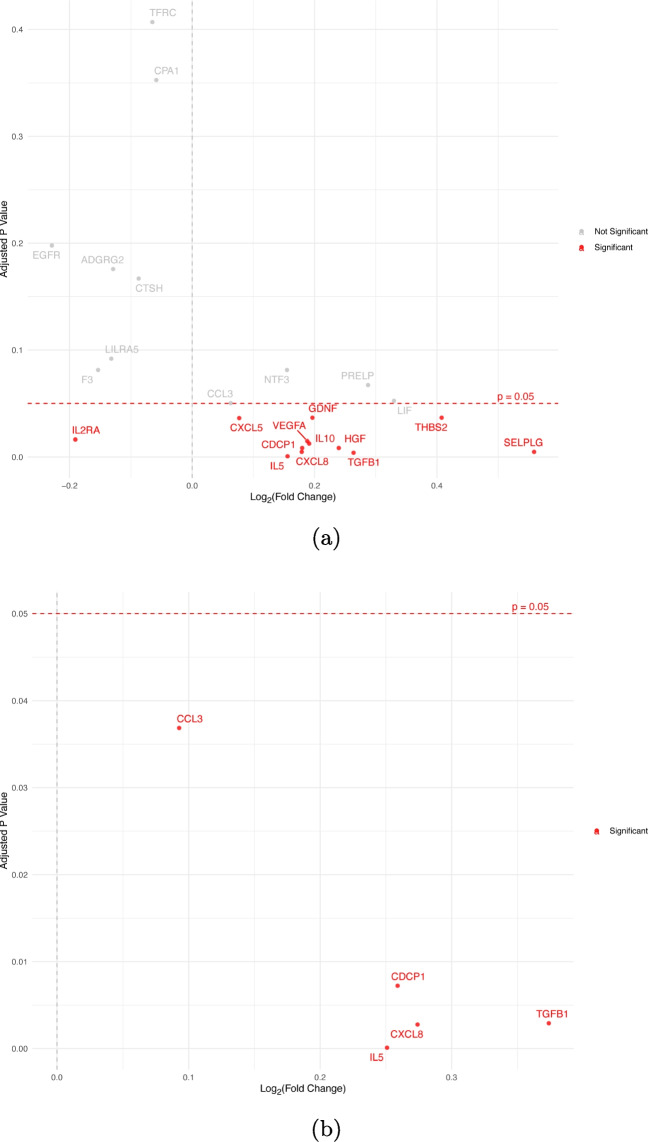
**Differential expression analysis of sleep duration categorized by sleep onset and wake-up times.** Proteins significantly dysregulated with a *p*-value of 0.05 after adjusting for multiple-testing with Benjamini-Hochberg false discovery rate (FDR) are colored in red. **a** Daytime sleep: Defined as sleep that begins after 09:00 and ends before 19:00. **b** Afternoon sleep: Defined as sleep that starts after noon and ends before 19:00

**Fig. 6:**
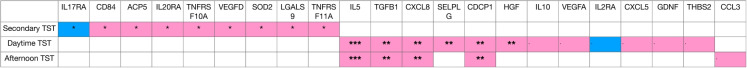
**Comparison of proteins dysregulated with different sleep patterns.** **a** Average secondary sleep TST. **b** Analysis based on the timing of sleep—daytime (09:00 to 19:00) TST and afternoon (12:00 to 19:00) TST. Pink and blue represent the up-regulation and down-regulation of protein abundance levels respectively. * $q < 0.05$, ** $q < 0.01$, *** $q < 0.001$, white background for non-significant assays

Similarly, LASSO regression reduced the number of proteins considered for differential expression analysis to 23 and 5 to be considered for daytime TST and afternoon TST, respectively. The subsequent differential expression analysis of secondary sleep based on the time of day revealed 12 assays to be associated with daytime TST and 5 assays to be associated with afternoon TST. The statistically significant assays ($q < 0.05$) for daytime and afternoon sleep are shown in red in Fig. 5. The complete list of proteins dysregulated with different sleep variables is depicted in Fig. 6. We also performed the same analysis for secondary sleep episodes that fall outside the defined daytime period but did not identify any significant protein assays.

## Discussion

This study explored the relationship between objectively measured sleep patterns and protein biomarkers in an elderly population. It is the first study to collect daily wearable device data for over a calendar year in naturalistic conditions among older adults. By analyzing sleep data from wearable devices alongside proteomic data, we focused specifically on secondary sleep—sleep episodes occurring outside the main sleep period within a day. We discovered that the secondary sleep TST is inversely correlated with the daily average main sleep TST. The proteomic analysis identified 9 proteins significantly associated with longer secondary sleep TST. Additionally, a further breakdown of the timing of these sleep episodes revealed that 12 proteins were linked to daytime TST, while 5 were associated with afternoon TST. The majority of the dysregulated proteins were associated with biological processes related to inflammatory and immune pathways.

This study provides insights into plasma protein variations associated with sleep behavior in the elderly with wearable devices. Our results revealed that most participants had secondary sleep episodes besides their main sleep.

Monitoring sleep solely through self-reported questionnaires is not feasible as it may overlook important patterns, especially with regard to involuntary or fragmented sleep.

**Table 2:** Biological processes enriched in association with increasing secondary sleep TST

Rank	Biological process	Proteins
1	**Negative regulation of immune effector process**	**CD84, ACP5, LGALS9**
2	**Positive regulation of chemokine production**	**IL17RA, CD84, LGALS9**

The functional enrichment analysis was conducted using Metascape

Terms with a *p*-value $< 0.01$, a minimum count of 3, and an enrichment factor $> 1.5$ were collected and grouped into clusters based on their membership similarities, with *p*-values calculated using the accumulative hypergeometric distribution

We discovered 9 plasma proteins that were dysregulated with increasing secondary sleep TSTs among participants. The significantly dysregulated proteins include cytokines belonging to the TNF superfamily—death receptor 4 (TNFRSF11A) and receptor activator of nuclear factor $\kappa$-B (TNFRSF10A, also known as RANK) protein. TNF receptors have been linked to a range of crucial activities such as inflammation [23], sleep regulation [45], and fatigue [46]. The dysregulation of proteins involved in immune and inflammatory regulation, including IL17RA, CD84, IL20RA, and TNFRSF10A, was particularly notable. This finding suggests that inflammation and immune responses may be critical in influencing sleep behavior. Previous studies have shown that cytokine production negatively regulates sleep-wake behavior [24] and sleep disruptions can affect the immune system in a reciprocal manner [15].

The analysis of protein abundances by the timing of sleep episodes revealed that 12 assays were associated with daytime sleep and 5 with afternoon sleep. Four proteins—Interleukin-5 (IL5), Interleukin-8 (CXCL8), Transforming Growth Factor Beta 1 (TGFB1), and CUB domain-containing protein 1 (CDCP1)—were up-regulated with both daytime and afternoon sleep times. Elevated CXCL8 levels have been associated with obstructive sleep apnea syndrome in children and adults [47]. However, the existing research literature does not indicate a direct connection between the rest of the proteins and sleep conditions. A previous study discovered Complement C1q tumor necrosis factor-related protein 1 (C1QTNF1) to be up-regulated in participants who suffered from daytime sleepiness [48]. However, their study has been limited to measuring only the C1QTNF1 protein and a single night of sleep data recorded with PSG.

Further analysis of the differentially expressed proteins using functional analysis revealed 2 biological processes to be statistically significant—negative regulation of the immune effector process and positive regulation of chemokine production.

Designing custom panels that target specific biochemical pathways involved in sleep regulation could enable the diagnosis of sleep disorders in a clinical setting. By measuring biomarkers directly linked to these pathways, such as those in the TNF superfamily and NF-$\textsc {k}$B signaling, healthcare professionals can obtain precise insights into the underlying mechanisms of a patient’s sleep disturbances and provide personalized feedback to improve sleep quality.

We acknowledge the limitations of this study, particularly the small sample size. Future research should address these limitations by involving larger and more diverse cohorts to validate and expand upon our findings. Despite adjusting for multiple potential confounders such as age, sex, and commonly measured clinical parameters, there may still be unmeasured variables such as the severity or specific types of chronic diseases like heart failure. Future studies that include detailed assessments of disease severity and additional clinical biomarkers may more thoroughly account for these confounding effects that could influence both protein expression and sleep patterns. Additionally, integrating broader omics platforms [49] such as genomics and metabolomics will enhance our understanding of the interplay between genetic, environmental, and lifestyle factors. With access to larger data sets and varied data modalities, we can utilize state-of-the-art machine learning approaches [50, 51] as another avenue for discovering novel biomarkers responsible for sleeping patterns in the elderly. A better understanding of biomarkers and how they impact biological pathways can be utilized to design clinical and environmental interventions that improve the sleep and overall health of the elderly.

This study has several strengths. First, it is the first study to measure 24-h activity and sleep for over a year in an elderly population using wearable devices. This comprehensive approach allowed us to capture detailed sleep patterns, including secondary sleep episodes, which are often overlooked in traditional studies. Second, by integrating objective sleep data with extensive proteomic analysis, we enhanced our understanding of the multifactorial nature of sleep disturbances in the elderly. This integration revealed specific protein biomarkers associated with secondary sleep and highlighted potential links to inflammatory and immune processes, providing valuable insights into age-related changes in sleep behavior.

## Ethical statement

All participants received detailed information about the purpose of the study during an explanatory face-to-face clinic visit. The nature of the research and details of the study have been explained to all participants in accordance with the documents approved by the Ethics Review Committee.

### The cohort study

This study was approved by the University of Tokyo ethics committee (approved number: KE23-57), and the research outline of this study is registered in the public database of the University Hospital Medical Information Network (UMIN).

## Supplementary Information

Below is the link to the electronic supplementary material.

Supplementary file 1 (pdf 113 KB)

## Data Availability

We cannot provide public access to individual data due to participant privacy stipulations in accordance with ethical guidelines. Additionally, the written informed consent we obtained from study participants does not include a provision for the public sharing of data.

## Footnotes

1 https://www.fitbit.com/global/us/products/trackers/inspire2

2 https://olink.com/products-services/target/

## Code availability

Code can be made available upon request by contacting the corresponding author.
