# Supplementary material for: Associations between plasma proteomic signatures and secondary sleep in older adults
Source: GeroScience. 2025 Apr 8;47(3):4623–34. doi: 10.1007/s11357-025-01565-1 (PMC12181523; doi:10.1007/s11357-025-01565-1)
Supplement: Supplementary file 1 — (pdf 113 KB) [file 11357_2025_1565_MOESM1_ESM.pdf]

# Plasma proteome profiling of sleep behavior in the elderly using wearable devices reveals biomarkers associated with inflammation

## 1 Proteomic Data

**Table 1**: Comprehensive details of the 355 Olink assays used in the analysis. Note that some proteins may be measured by multiple assays across different panels.

| Row | OlinkID | Assay | UniProt | Panel |
|---|---|---|---|---|
| 0 | OID00132 | MEPE | Q9NQ76 | Olink Cardiovascular III |
| 1 | OID00379 | BMP6 | P22004 | Olink Cardiovascular II |
| 2 | OID00380 | ANGPT1 | Q15389 | Olink Cardiovascular II |
| 3 | OID00381 | ADM | P35318 | Olink Cardiovascular II |
| 4 | OID00382 | CD40LG | P29965 | Olink Cardiovascular II |
| 5 | OID00383 | SLAMF7 | Q9NQ25 | Olink Cardiovascular II |
| 6 | OID00384 | PGF | P49763 | Olink Cardiovascular II |
| 7 | OID00385 | ADAMTS13 | Q76LX8 | Olink Cardiovascular II |
| 8 | OID00386 | BOC | Q9BWV1 | Olink Cardiovascular II |
| 9 | OID00387 | IL4R | P24394 | Olink Cardiovascular II |
| 10 | OID00388 | SRC | P12931 | Olink Cardiovascular II |
| 11 | OID00389 | IL1RN | P18510 | Olink Cardiovascular II |
| 12 | OID00390 | IL6 | P05231 | Olink Cardiovascular II |
| 13 | OID00391 | TNFRSF10A | O00220 | Olink Cardiovascular II |
| 14 | OID00392 | STK4 | Q13043 | Olink Cardiovascular II |
| 15 | OID00393 | IDUA | P35475 | Olink Cardiovascular II |
| 16 | OID00394 | TNFRSF11A | Q9Y6Q6 | Olink Cardiovascular II |
| 17 | OID00395 | F2R | P25116 | Olink Cardiovascular II |
| 18 | OID00396 | TNFRSF10B | O14763 | Olink Cardiovascular II |
| 19 | OID00397 | PRSS27 | Q9BQR3 | Olink Cardiovascular II |
| 20 | OID00398 | TEK | Q02763 | Olink Cardiovascular II |
| 21 | OID00399 | F3 | P13726 | Olink Cardiovascular II |

Continued on next page

| Row | OlinkID | Assay | UniProt | Panel |
|---|---|---|---|---|
| 22 | OID00400 | IL1RL2 | Q9HB29 | Olink Cardiovascular II |
| 23 | OID00401 | PDGFB | P01127 | Olink Cardiovascular II |
| 24 | OID00403 | IL17D | Q8TAD2 | Olink Cardiovascular II |
| 25 | OID00404 | CXCL1 | P09341 | Olink Cardiovascular II |
| 26 | OID00405 | OLR1 | P78380 | Olink Cardiovascular II |
| 27 | OID00406 | LGALS9 | O00182 | Olink Cardiovascular II |
| 28 | OID00407 | CBLIF | P27352 | Olink Cardiovascular II |
| 29 | OID00408 | KITLG | P21583 | Olink Cardiovascular II |
| 30 | OID00409 | IL18 | Q14116 | Olink Cardiovascular II |
| 31 | OID00410 | FGF21 | Q9NSA1 | Olink Cardiovascular II |
| 32 | OID00411 | PIGR | P01833 | Olink Cardiovascular II |
| 33 | OID00412 | AGER | Q15109 | Olink Cardiovascular II |
| 34 | OID00413 | SOD2 | P04179 | Olink Cardiovascular II |
| 35 | OID00414 | CTRC | Q99895 | Olink Cardiovascular II |
| 36 | OID00415 | FGF23 | Q9GZV9 | Olink Cardiovascular II |
| 37 | OID00416 | SPON2 | Q9BUD6 | Olink Cardiovascular II |
| 38 | OID00417 | GH1 | P01241 | Olink Cardiovascular II |
| 39 | OID00418 | FST | P19883 | Olink Cardiovascular II |
| 40 | OID00419 | GLO1 | Q04760 | Olink Cardiovascular II |
| 41 | OID00420 | CD84 | Q9UIB8 | Olink Cardiovascular II |
| 42 | OID00421 | PAPPA | Q13219 | Olink Cardiovascular II |
| 43 | OID00422 | SERPINA12 | Q8IW75 | Olink Cardiovascular II |
| 44 | OID00423 | REN | P00797 | Olink Cardiovascular II |
| 45 | OID00424 | DECR1 | Q16698 | Olink Cardiovascular II |
| 46 | OID00425 | MERTK | Q12866 | Olink Cardiovascular II |
| 47 | OID00426 | HAVCR1 | Q96D42 | Olink Cardiovascular II |
| 48 | OID00427 | THBS2 | P35442 | Olink Cardiovascular II |
| 49 | OID00428 | THBD | P07204 | Olink Cardiovascular II |
| 50 | OID00429 | VSIG2 | Q96IQ7 | Olink Cardiovascular II |
| 51 | OID00430 | AMBP | P02760 | Olink Cardiovascular II |
| 52 | OID00431 | PRELP | P51888 | Olink Cardiovascular II |
| 53 | OID00432 | HMOX1 | P09601 | Olink Cardiovascular II |
| 54 | OID00433 | XCL1 | P47992 | Olink Cardiovascular II |
| 55 | OID00434 | IL16 | Q14005 | Olink Cardiovascular II |
| 56 | OID00435 | SORT1 | Q99523 | Olink Cardiovascular II |
| 57 | OID00436 | CEACAM8 | P31997 | Olink Cardiovascular II |
| 58 | OID00437 | PTX3 | P26022 | Olink Cardiovascular II |
| 59 | OID00438 | SELPLG | Q14242 | Olink Cardiovascular II |
| 60 | OID00439 | CCL17 | Q92583 | Olink Cardiovascular II |
| 61 | OID00440 | CCL3 | P10147 | Olink Cardiovascular II |
| 62 | OID00441 | MMP7 | P09237 | Olink Cardiovascular II |
| 63 | OID00442 | FCGR2B | P31994 | Olink Cardiovascular II |

Continued on next page

| Row | OlinkID | Assay | UniProt | Panel |
|---|---|---|---|---|
| 64 | OID00443 | ITGB1BP2 | Q9UKP3 | Olink Cardiovascular II |
| 65 | OID00444 | DCN | P07585 | Olink Cardiovascular II |
| 66 | OID00445 | DKK1 | O94907 | Olink Cardiovascular II |
| 67 | OID00446 | LPL | P06858 | Olink Cardiovascular II |
| 68 | OID00447 | PRSS8 | Q16651 | Olink Cardiovascular II |
| 69 | OID00448 | AGRP | O00253 | Olink Cardiovascular II |
| 70 | OID00449 | HBEGF | Q99075 | Olink Cardiovascular II |
| 71 | OID00450 | GDF2 | Q9UK05 | Olink Cardiovascular II |
| 72 | OID00451 | FABP2 | P12104 | Olink Cardiovascular II |
| 73 | OID00452 | THPO | P40225 | Olink Cardiovascular II |
| 74 | OID00453 | MARCO | Q9UEW3 | Olink Cardiovascular II |
| 75 | OID00454 | FABP6 | P51161 | Olink Cardiovascular II |
| 76 | OID00455 | NPPB | P16860 | Olink Cardiovascular II |
| 77 | OID00456 | MMP12 | P39900 | Olink Cardiovascular II |
| 78 | OID00457 | ACE2 | Q9BYF1 | Olink Cardiovascular II |
| 79 | OID00458 | PDCD1LG2 | Q9BQ51 | Olink Cardiovascular II |
| 80 | OID00459 | CTSL | P07711 | Olink Cardiovascular II |
| 81 | OID00460 | OSCAR | Q8IYS5 | Olink Cardiovascular II |
| 82 | OID00461 | TNFRSF13B | O14836 | Olink Cardiovascular II |
| 83 | OID00462 | TGM2 | P21980 | Olink Cardiovascular II |
| 84 | OID00463 | LEP | P41159 | Olink Cardiovascular II |
| 85 | OID00464 | CA5A | P35218 | Olink Cardiovascular II |
| 86 | OID00465 | HSPB1 | P04792 | Olink Cardiovascular II |
| 87 | OID00466 | CD4 | P01730 | Olink Cardiovascular II |
| 88 | OID00467 | IKBKG | Q9Y6K9 | Olink Cardiovascular II |
| 89 | OID00468 | VEGFD | O43915 | Olink Cardiovascular II |
| 90 | OID00469 | PARP1 | P09874 | Olink Cardiovascular II |
| 91 | OID00470 | HAO1 | Q9UJM8 | Olink Cardiovascular II |
| 92 | OID00471 | CXCL8 | P10145 | Olink Inflammation |
| 93 | OID00472 | VEGFA | P15692 | Olink Inflammation |
| 94 | OID00474 | CCL7 | P80098 | Olink Inflammation |
| 95 | OID00475 | GDNF | P39905 | Olink Inflammation |
| 96 | OID00476 | CDCP1 | Q9H5V8 | Olink Inflammation |
| 97 | OID00477 | CD244 | Q9BZW8 | Olink Inflammation |
| 98 | OID00478 | IL7 | P13232 | Olink Inflammation |
| 99 | OID00479 | TNFRSF11B | O00300 | Olink Inflammation |
| 100 | OID00480 | TGFB1 | P01137 | Olink Inflammation |
| 101 | OID00481 | PLAU | P00749 | Olink Inflammation |
| 102 | OID00482 | IL6 | P05231 | Olink Inflammation |
| 103 | OID00483 | IL17C | Q9P0M4 | Olink Inflammation |
| 104 | OID00484 | CCL2 | P13500 | Olink Inflammation |
| 105 | OID00485 | IL17A | Q16552 | Olink Inflammation |

Continued on next page

| Row | OlinkID | Assay | UniProt | Panel |
|---|---|---|---|---|
| 106 | OID00486 | CXCL11 | O14625 | Olink Inflammation |
| 107 | OID00487 | AXIN1 | O15169 | Olink Inflammation |
| 108 | OID00488 | TNFSF10 | P50591 | Olink Inflammation |
| 109 | OID00489 | IL20RA | Q9UHF4 | Olink Inflammation |
| 110 | OID00490 | CXCL9 | Q07325 | Olink Inflammation |
| 111 | OID00491 | CST5 | P28325 | Olink Inflammation |
| 112 | OID00492 | IL2RB | P14784 | Olink Inflammation |
| 113 | OID00493 | IL1A | P01583 | Olink Inflammation |
| 114 | OID00494 | OSM | P13725 | Olink Inflammation |
| 115 | OID00495 | IL2 | P60568 | Olink Inflammation |
| 116 | OID00496 | CXCL1 | P09341 | Olink Inflammation |
| 117 | OID00497 | TSLP | Q969D9 | Olink Inflammation |
| 118 | OID00498 | CCL4 | P13236 | Olink Inflammation |
| 119 | OID00499 | CD6 | P30203 | Olink Inflammation |
| 120 | OID00500 | KITLG | P21583 | Olink Inflammation |
| 121 | OID00501 | IL18 | Q14116 | Olink Inflammation |
| 122 | OID00502 | SLAMF1 | Q13291 | Olink Inflammation |
| 123 | OID00503 | TGFA | P01135 | Olink Inflammation |
| 124 | OID00504 | CCL13 | Q99616 | Olink Inflammation |
| 125 | OID00505 | CCL11 | P51671 | Olink Inflammation |
| 126 | OID00506 | TNFSF14 | O43557 | Olink Inflammation |
| 127 | OID00507 | FGF23 | Q9GZV9 | Olink Inflammation |
| 128 | OID00508 | IL10RA | Q13651 | Olink Inflammation |
| 129 | OID00509 | FGF5 | P12034 | Olink Inflammation |
| 130 | OID00510 | MMP1 | P03956 | Olink Inflammation |
| 131 | OID00511 | LIFR | P42702 | Olink Inflammation |
| 132 | OID00512 | FGF21 | Q9NSA1 | Olink Inflammation |
| 133 | OID00513 | CCL19 | Q99731 | Olink Inflammation |
| 134 | OID00514 | IL15RA | Q13261 | Olink Inflammation |
| 135 | OID00515 | IL10RB | Q08334 | Olink Inflammation |
| 136 | OID00516 | IL22RA1 | Q8N6P7 | Olink Inflammation |
| 137 | OID00517 | IL18R1 | Q13478 | Olink Inflammation |
| 138 | OID00518 | CD274 | Q9NZQ7 | Olink Inflammation |
| 139 | OID00519 | NGF | P01138 | Olink Inflammation |
| 140 | OID00520 | CXCL5 | P42830 | Olink Inflammation |
| 141 | OID00521 | TNFSF11 | O14788 | Olink Inflammation |
| 142 | OID00522 | HGF | P14210 | Olink Inflammation |
| 143 | OID00523 | IL12B | P29460 | Olink Inflammation |
| 144 | OID00524 | IL24 | Q13007 | Olink Inflammation |
| 145 | OID00525 | IL13 | P35225 | Olink Inflammation |
| 146 | OID00526 | ARTN | Q5T4W7 | Olink Inflammation |
| 147 | OID00527 | MMP10 | P09238 | Olink Inflammation |

Continued on next page

| Row | OlinkID | Assay | UniProt | Panel |
|---|---|---|---|---|
| 148 | OID00528 | IL10 | P22301 | Olink Inflammation |
| 149 | OID00530 | CCL23 | P55773 | Olink Inflammation |
| 150 | OID00531 | CD5 | P06127 | Olink Inflammation |
| 151 | OID00532 | CCL3 | P10147 | Olink Inflammation |
| 152 | OID00533 | FLT3LG | P49771 | Olink Inflammation |
| 153 | OID00534 | CXCL6 | P80162 | Olink Inflammation |
| 154 | OID00535 | CXCL10 | P02778 | Olink Inflammation |
| 155 | OID00536 | EIF4EBP1 | Q13541 | Olink Inflammation |
| 156 | OID00537 | IL20 | Q9NYY1 | Olink Inflammation |
| 157 | OID00538 | SIRT2 | Q8IXJ6 | Olink Inflammation |
| 158 | OID00539 | CCL28 | Q9NRJ3 | Olink Inflammation |
| 159 | OID00541 | S100A12 | P80511 | Olink Inflammation |
| 160 | OID00542 | CD40 | P25942 | Olink Inflammation |
| 161 | OID00543 | IL33 | O95760 | Olink Inflammation |
| 162 | OID00545 | FGF19 | O95750 | Olink Inflammation |
| 163 | OID00546 | IL4 | P05112 | Olink Inflammation |
| 164 | OID00547 | LIF | P15018 | Olink Inflammation |
| 165 | OID00548 | NRTN | Q99748 | Olink Inflammation |
| 166 | OID00549 | CCL8 | P80075 | Olink Inflammation |
| 167 | OID00550 | CASP8 | Q14790 | Olink Inflammation |
| 168 | OID00551 | CCL25 | O15444 | Olink Inflammation |
| 169 | OID00552 | CX3CL1 | P78423 | Olink Inflammation |
| 170 | OID00553 | TNFRSF9 | Q07011 | Olink Inflammation |
| 171 | OID00554 | NTF3 | P20783 | Olink Inflammation |
| 172 | OID00555 | TNFSF12 | O43508 | Olink Inflammation |
| 173 | OID00556 | CCL20 | P78556 | Olink Inflammation |
| 174 | OID00557 | SULT1A1 | P50225 | Olink Inflammation |
| 175 | OID00558 | STAMBP | O95630 | Olink Inflammation |
| 176 | OID00559 | IL5 | P05113 | Olink Inflammation |
| 177 | OID00560 | ADA | P00813 | Olink Inflammation |
| 178 | OID00561 | LTA | P01374 | Olink Inflammation |
| 179 | OID00562 | CSF1 | P09603 | Olink Inflammation |
| 180 | OID00563 | TNFRSF14 | Q92956 | Olink Cardiovascular III |
| 181 | OID00564 | LDLR | P01130 | Olink Cardiovascular III |
| 182 | OID00565 | ITGB2 | P05107 | Olink Cardiovascular III |
| 183 | OID00566 | IL17RA | Q96F46 | Olink Cardiovascular III |
| 184 | OID00567 | TNFRSF1B | P20333 | Olink Cardiovascular III |
| 185 | OID00568 | MMP9 | P14780 | Olink Cardiovascular III |
| 186 | OID00569 | EPHB4 | P54760 | Olink Cardiovascular III |
| 187 | OID00570 | IL2RA | P01589 | Olink Cardiovascular III |
| 188 | OID00571 | TNFRSF11B | O00300 | Olink Cardiovascular III |
| 189 | OID00572 | ALCAM | Q13740 | Olink Cardiovascular III |

Continued on next page

| Row | OlinkID | Assay | UniProt | Panel |
|---|---|---|---|---|
| 190 | OID00573 | TFF3 | Q07654 | Olink Cardiovascular III |
| 191 | OID00574 | SELP | P16109 | Olink Cardiovascular III |
| 192 | OID00575 | CSTB | P04080 | Olink Cardiovascular III |
| 193 | OID00576 | CCL2 | P13500 | Olink Cardiovascular III |
| 194 | OID00577 | CD163 | Q86VB7 | Olink Cardiovascular III |
| 195 | OID00578 | LGALS3 | P17931 | Olink Cardiovascular III |
| 196 | OID00579 | GRN | P28799 | Olink Cardiovascular III |
| 197 | OID00581 | BLMH | Q13867 | Olink Cardiovascular III |
| 198 | OID00582 | HSPG2 | P98160 | Olink Cardiovascular III |
| 199 | OID00583 | LTBR | P36941 | Olink Cardiovascular III |
| 200 | OID00584 | NOTCH3 | Q9UM47 | Olink Cardiovascular III |
| 201 | OID00585 | TIMP4 | Q99727 | Olink Cardiovascular III |
| 202 | OID00586 | CNTN1 | Q12860 | Olink Cardiovascular III |
| 203 | OID00587 | CDH5 | P33151 | Olink Cardiovascular III |
| 204 | OID00588 | TREML2 | Q5T2D2 | Olink Cardiovascular III |
| 205 | OID00589 | FABP4 | P15090 | Olink Cardiovascular III |
| 206 | OID00590 | TFPI | P10646 | Olink Cardiovascular III |
| 207 | OID00591 | SERPINE1 | P05121 | Olink Cardiovascular III |
| 208 | OID00592 | CCL24 | O00175 | Olink Cardiovascular III |
| 209 | OID00593 | TFRC | P02786 | Olink Cardiovascular III |
| 210 | OID00594 | TNFRSF10C | O14798 | Olink Cardiovascular III |
| 211 | OID00595 | GDF15 | Q99988 | Olink Cardiovascular III |
| 212 | OID00596 | SELE | P16581 | Olink Cardiovascular III |
| 213 | OID00597 | AZU1 | P20160 | Olink Cardiovascular III |
| 214 | OID00598 | DLK1 | P80370 | Olink Cardiovascular III |
| 215 | OID00599 | SPON1 | Q9HCB6 | Olink Cardiovascular III |
| 216 | OID00600 | MPO | P05164 | Olink Cardiovascular III |
| 217 | OID00601 | CXCL16 | Q9H2A7 | Olink Cardiovascular III |
| 218 | OID00602 | IL6R | P08887 | Olink Cardiovascular III |
| 219 | OID00603 | RETN | Q9HD89 | Olink Cardiovascular III |
| 220 | OID00604 | IGFBP1 | P08833 | Olink Cardiovascular III |
| 221 | OID00605 | CHIT1 | Q13231 | Olink Cardiovascular III |
| 222 | OID00606 | ACP5 | P13686 | Olink Cardiovascular III |
| 223 | OID00608 | SFTPD | P35247 | Olink Cardiovascular III |
| 224 | OID00609 | PI3 | P19957 | Olink Cardiovascular III |
| 225 | OID00610 | EPCAM | P16422 | Olink Cardiovascular III |
| 226 | OID00611 | ANPEP | P15144 | Olink Cardiovascular III |
| 227 | OID00612 | AXL | P30530 | Olink Cardiovascular III |
| 228 | OID00613 | IL1R1 | P14778 | Olink Cardiovascular III |
| 229 | OID00614 | MMP2 | P08253 | Olink Cardiovascular III |
| 230 | OID00615 | FAS | P25445 | Olink Cardiovascular III |
| 231 | OID00616 | MB | P02144 | Olink Cardiovascular III |

Continued on next page

| Row | OlinkID | Assay | UniProt | Panel |
|---|---|---|---|---|
| 232 | OID00617 | TNFSF13B | Q9Y275 | Olink Cardiovascular III |
| 233 | OID00618 | PRTN3 | P24158 | Olink Cardiovascular III |
| 234 | OID00619 | PCSK9 | Q8NBP7 | Olink Cardiovascular III |
| 235 | OID00620 | PLAUR | Q03405 | Olink Cardiovascular III |
| 236 | OID00621 | SPP1 | P10451 | Olink Cardiovascular III |
| 237 | OID00622 | CTSD | P07339 | Olink Cardiovascular III |
| 238 | OID00623 | PGLYRP1 | O75594 | Olink Cardiovascular III |
| 239 | OID00624 | CPA1 | P15085 | Olink Cardiovascular III |
| 240 | OID00625 | F11R | Q9Y624 | Olink Cardiovascular III |
| 241 | OID00626 | LGALS4 | P56470 | Olink Cardiovascular III |
| 242 | OID00627 | IL1R2 | P27930 | Olink Cardiovascular III |
| 243 | OID00628 | SIRPA | P78324 | Olink Cardiovascular III |
| 244 | OID00629 | CCL15 | Q16663 | Olink Cardiovascular III |
| 245 | OID00630 | CASP3 | P42574 | Olink Cardiovascular III |
| 246 | OID00631 | PLAU | P00749 | Olink Cardiovascular III |
| 247 | OID00632 | CPB1 | P15086 | Olink Cardiovascular III |
| 248 | OID00633 | CHI3L1 | P36222 | Olink Cardiovascular III |
| 249 | OID00634 | IL1RL1 | Q01638 | Olink Cardiovascular III |
| 250 | OID00635 | PLAT | P00750 | Olink Cardiovascular III |
| 251 | OID00636 | SCGB3A2 | Q96PL1 | Olink Cardiovascular III |
| 252 | OID00637 | EGFR | P00533 | Olink Cardiovascular III |
| 253 | OID00638 | IGFBP7 | Q16270 | Olink Cardiovascular III |
| 254 | OID00639 | CD93 | Q9NPY3 | Olink Cardiovascular III |
| 255 | OID00640 | IL18BP | O95998 | Olink Cardiovascular III |
| 256 | OID00641 | COL1A1 | P02452 | Olink Cardiovascular III |
| 257 | OID00642 | PON3 | Q15166 | Olink Cardiovascular III |
| 258 | OID00643 | CTSZ | Q9UBR2 | Olink Cardiovascular III |
| 259 | OID00644 | MMP3 | P08254 | Olink Cardiovascular III |
| 260 | OID00645 | RARRES2 | Q99969 | Olink Cardiovascular III |
| 261 | OID00646 | ICAM2 | P13598 | Olink Cardiovascular III |
| 262 | OID00647 | KLK6 | Q92876 | Olink Cardiovascular III |
| 263 | OID00648 | PDGFA | P04085 | Olink Cardiovascular III |
| 264 | OID00649 | TNFRSF1A | P19438 | Olink Cardiovascular III |
| 265 | OID00650 | IGFBP2 | P18065 | Olink Cardiovascular III |
| 266 | OID00651 | VWF | P04275 | Olink Cardiovascular III |
| 267 | OID00652 | PECAM1 | P16284 | Olink Cardiovascular III |
| 268 | OID00654 | CCL16 | O15467 | Olink Cardiovascular III |
| 269 | OID01120 | CLMP | Q9H6B4 | Olink Metabolism |
| 270 | OID01121 | LRIG1 | Q96JA1 | Olink Metabolism |
| 271 | OID01122 | NPTXR | O95502 | Olink Metabolism |
| 272 | OID01123 | AHCY | P23526 | Olink Metabolism |
| 273 | OID01124 | THOP1 | P52888 | Olink Metabolism |

Continued on next page

| Row | OlinkID | Assay | UniProt | Panel |
|---|---|---|---|---|
| 274 | OID01125 | CTSO | P43234 | Olink Metabolism |
| 275 | OID01126 | FCRL1 | Q96LA6 | Olink Metabolism |
| 276 | OID01127 | CD164 | Q04900 | Olink Metabolism |
| 277 | OID01128 | DDC | P20711 | Olink Metabolism |
| 278 | OID01129 | ACP6 | Q9NPH0 | Olink Metabolism |
| 279 | OID01130 | TFF2 | Q03403 | Olink Metabolism |
| 280 | OID01131 | S100P | P25815 | Olink Metabolism |
| 281 | OID01132 | ANGPT2 | O15123 | Olink Metabolism |
| 282 | OID01133 | CD2AP | Q9Y5K6 | Olink Metabolism |
| 283 | OID01134 | ANGPTL7 | O43827 | Olink Metabolism |
| 284 | OID01135 | CLEC5A | Q9NY25 | Olink Metabolism |
| 285 | OID01136 | TINAGL1 | Q9GZM7 | Olink Metabolism |
| 286 | OID01137 | GLRX | P35754 | Olink Metabolism |
| 287 | OID01138 | ENO2 | P09104 | Olink Metabolism |
| 288 | OID01139 | NADK | O95544 | Olink Metabolism |
| 289 | OID01140 | GHRL | Q9UBU3 | Olink Metabolism |
| 290 | OID01141 | SERPINB8 | P50452 | Olink Metabolism |
| 291 | OID01142 | SERPINB6 | P35237 | Olink Metabolism |
| 292 | OID01143 | CDHR5 | Q9HBB8 | Olink Metabolism |
| 293 | OID01144 | CCDC80 | Q76M96 | Olink Metabolism |
| 294 | OID01145 | DIABLO | Q9NR28 | Olink Metabolism |
| 295 | OID01146 | CA13 | Q8N1Q1 | Olink Metabolism |
| 296 | OID01147 | SEMA3F | Q13275 | Olink Metabolism |
| 297 | OID01148 | KLK10 | O43240 | Olink Metabolism |
| 298 | OID01149 | PILRB | Q9UKJ0 | Olink Metabolism |
| 299 | OID01150 | ANGPTL1 | O95841 | Olink Metabolism |
| 300 | OID01151 | APLP1 | P51693 | Olink Metabolism |
| 301 | OID01152 | ADGRG2 | Q8IZP9 | Olink Metabolism |
| 302 | OID01153 | TYMP | P19971 | Olink Metabolism |
| 303 | OID01154 | GRAP2 | O75791 | Olink Metabolism |
| 304 | OID01155 | LILRA5 | A6NI73 | Olink Metabolism |
| 305 | OID01156 | ALDH1A1 | P00352 | Olink Metabolism |
| 306 | OID01157 | CD79B | P40259 | Olink Metabolism |
| 307 | OID01158 | ANXA4 | P09525 | Olink Metabolism |
| 308 | OID01159 | ANXA11 | P50995 | Olink Metabolism |
| 309 | OID01160 | SIGLEC7 | Q9Y286 | Olink Metabolism |
| 310 | OID01161 | ITGB7 | P26010 | Olink Metabolism |
| 311 | OID01162 | QDPR | P09417 | Olink Metabolism |
| 312 | OID01163 | SNAP23 | O00161 | Olink Metabolism |
| 313 | OID01164 | APEX1 | P27695 | Olink Metabolism |
| 314 | OID01165 | ENTPD5 | O75356 | Olink Metabolism |
| 315 | OID01166 | CLSTN2 | Q9H4D0 | Olink Metabolism |

Continued on next page

| Row | OlinkID | Assay | UniProt | Panel |
|---|---|---|---|---|
| 316 | OID01167 | COMT | P21964 | Olink Metabolism |
| 317 | OID01168 | CLUL1 | Q15846 | Olink Metabolism |
| 318 | OID01169 | HDGF | P51858 | Olink Metabolism |
| 319 | OID01170 | CHRDL2 | Q6WN34 | Olink Metabolism |
| 320 | OID01171 | CTSH | P09668 | Olink Metabolism |
| 321 | OID01172 | NOMO1 | Q15155 | Olink Metabolism |
| 322 | OID01173 | NQO2 | P16083 | Olink Metabolism |
| 323 | OID01174 | SOST | Q9BQB4 | Olink Metabolism |
| 324 | OID01175 | FAM3C | Q92520 | Olink Metabolism |
| 325 | OID01176 | TXNDC5 | Q8NBS9 | Olink Metabolism |
| 326 | OID01177 | PPP1R2 | P41236 | Olink Metabolism |
| 327 | OID01178 | DPP7 | Q9UHL4 | Olink Metabolism |
| 328 | OID01179 | LRP11 | Q86VZ4 | Olink Metabolism |
| 329 | OID01180 | ADGRE2 | Q9UHX3 | Olink Metabolism |
| 330 | OID01181 | ENPP7 | Q6UWV6 | Olink Metabolism |
| 331 | OID01182 | SSC4D | Q8WTU2 | Olink Metabolism |
| 332 | OID01183 | MCFD2 | Q8NI22 | Olink Metabolism |
| 333 | OID01184 | REG4 | Q9BYZ8 | Olink Metabolism |
| 334 | OID01185 | SUMF2 | Q8NBJ7 | Olink Metabolism |
| 335 | OID01186 | CANT1 | Q8WVQ1 | Olink Metabolism |
| 336 | OID01187 | CD1C | P29017 | Olink Metabolism |
| 337 | OID01188 | GAL | P22466 | Olink Metabolism |
| 338 | OID01189 | CDH2 | P19022 | Olink Metabolism |
| 339 | OID01190 | TYRO3 | Q06418 | Olink Metabolism |
| 340 | OID01191 | CRKL | P46109 | Olink Metabolism |
| 341 | OID01192 | IGFBPL1 | Q8WX77 | Olink Metabolism |
| 342 | OID01193 | RTN4R | Q9BZR6 | Olink Metabolism |
| 343 | OID01194 | VCAN | P13611 | Olink Metabolism |
| 344 | OID01195 | FBP1 | P09467 | Olink Metabolism |
| 345 | OID01196 | TSHB | P01222 | Olink Metabolism |
| 346 | OID01197 | BAG6 | P46379 | Olink Metabolism |
| 347 | OID01198 | NECTIN2 | Q92692 | Olink Metabolism |
| 348 | OID01199 | ARG1 | P05089 | Olink Metabolism |
| 349 | OID01200 | USP8 | P40818 | Olink Metabolism |
| 350 | OID01201 | FKBP4 | Q02790 | Olink Metabolism |
| 351 | OID01202 | SDC4 | P31431 | Olink Metabolism |
| 352 | OID01203 | PAG1 | Q9NWQ8 | Olink Metabolism |
| 353 | OID01204 | KYAT1 | Q16773 | Olink Metabolism |
| 354 | OID01205 | DAB2 | P98082 | Olink Metabolism |
| 355 | OID01206 | NPDC1 | Q9NQX5 | Olink Metabolism |
| 356 | OID01207 | METRNL | Q641Q3 | Olink Metabolism |
| 357 | OID01208 | MEP1B | Q16820 | Olink Metabolism |

Continued on next page

| Row | OlinkID | Assay | UniProt | Panel |
|---|---|---|---|---|
| 358 | OID01209 | ROR1 | Q01973 | Olink Metabolism |
| 359 | OID01211 | RNASE3 | P12724 | Olink Metabolism |
| 360 | OID01213 | DNER | Q8NFT8 | Olink Inflammation |
| 361 | OID05026 | GP6 | Q9HCN6 | Olink Cardiovascular III |
| 362 | OID05124 | CD8A | P01732 | Olink Inflammation |
| 363 | OID05547 | IFNG | P01579 | Olink Inflammation |
| 364 | OID05548 | TNF | P01375 | Olink Inflammation |

# 2 Analysis

## 2.1 LASSO Regression Model

**Table 2**: Non-zero coefficients of the LASSO regression model fit to predict the secondary sleep TST.

| Variable | Coefficient | Abs(coefficient) |
|---|---|---|
| (Intercept) | 6.1002 | 6.1002 |
| TNFRSF11A | 1.5194 | 1.5194 |
| IL17RA | -1.3314 | 1.3314 |
| ACP5 | 0.9117 | 0.9117 |
| TNFRSF10A | 0.9056 | 0.9056 |
| fb_steps_mean | -0.7644 | 0.7644 |
| CPA1 | -0.5374 | 0.5374 |
| IL20RA | 0.5272 | 0.5272 |
| VEGFD | 0.4780 | 0.4780 |
| IL5 | 0.3710 | 0.3710 |
| LGALS9 | 0.2703 | 0.2703 |
| OSCAR | 0.1963 | 0.1963 |
| SOD2 | 0.1938 | 0.1938 |
| PLAT | 0.1331 | 0.1331 |
| CXCL5 | 0.1147 | 0.1147 |
| IL1RL2 | 0.0914 | 0.0914 |
| CD84 | 0.0532 | 0.0532 |

## 2.2 Differential Expression Analysis

**Table 3**: Differential expression analysis for secondary sleep TST. Statistically significant assays are highlighted in **bold**.

| Unnamed: 0 | Assay | logFC | AveExpr | t | P | Adjusted P | B |
|---|---|---|---|---|---|---|---|
| 1 | **IL17RA** | -1.008 | -0.000 | -2.983 | 0.004 | 0.041 | -2.051 |
| 2 | **CD84** | 0.883 | -0.000 | 2.810 | 0.007 | 0.041 | -2.455 |
| 3 | **ACP5** | 1.077 | 0.000 | 2.558 | 0.013 | 0.044 | -3.032 |
| 4 | **IL20RA** | 0.550 | 0.000 | 2.364 | 0.022 | 0.044 | -3.440 |
| 5 | **TNFRSF10A** | 0.701 | 0.000 | 2.356 | 0.022 | 0.044 | -3.439 |
| 6 | **VEGFD** | 1.170 | 0.000 | 2.318 | 0.024 | 0.044 | -3.515 |
| 7 | **SOD2** | 2.614 | 0.000 | 2.291 | 0.026 | 0.044 | -3.570 |
| 8 | **LGALS9** | 0.785 | 0.000 | 2.208 | 0.031 | 0.047 | -3.731 |
| 9 | **TNFRSF11A** | 0.404 | -0.000 | 2.150 | 0.036 | 0.048 | -3.841 |
| 10 | OSCAR | 0.712 | 0.000 | 1.583 | 0.119 | 0.143 | -4.781 |
| 11 | IL5 | 0.214 | 0.000 | 1.285 | 0.204 | 0.223 | -5.196 |
| 12 | CPA1 | -0.226 | -0.000 | -0.893 | 0.376 | 0.376 | -5.593 |

**Table 4**: Differential expression analysis for daytime sleep TST between 09:00 and 19:00.

| Unnamed: 0 | Assay | logFC | AveExpr | t | P | Adjusted P | B |
|---|---|---|---|---|---|---|---|
| 1 | **IL5** | 0.16 | 0.00 | 4.51 | 0.00 | 0.00 | 2.13 |
| 2 | **TGFB1** | 0.26 | 0.00 | 3.81 | 0.00 | 0.00 | -0.09 |
| 3 | **CXCL8** | 0.18 | -0.00 | 3.60 | 0.00 | 0.00 | -0.69 |
| 4 | **SELPLG** | 0.56 | -0.00 | 3.53 | 0.00 | 0.00 | -0.91 |
| 5 | **CDCP1** | 0.18 | -0.00 | 3.23 | 0.00 | 0.01 | -1.74 |
| 6 | **HGF** | 0.24 | 0.00 | 3.21 | 0.00 | 0.01 | -1.80 |
| 7 | **IL10** | 0.19 | 0.00 | 3.02 | 0.00 | 0.01 | -2.30 |
| 8 | **VEGFA** | 0.19 | 0.00 | 2.91 | 0.01 | 0.01 | -2.57 |
| 9 | **IL2RA** | -0.19 | -0.00 | -2.83 | 0.01 | 0.02 | -2.76 |
| 10 | **CXCL5** | 0.08 | -0.00 | 2.49 | 0.02 | 0.04 | -3.57 |
| 11 | **GDNF** | 0.20 | 0.00 | 2.42 | 0.02 | 0.04 | -3.73 |
| 12 | **THBS2** | 0.41 | 0.00 | 2.41 | 0.02 | 0.04 | -3.73 |
| 13 | CCL3 | 0.06 | 0.79 | 2.25 | 0.03 | 0.05 | -4.08 |
| 14 | LIF | 0.33 | 0.00 | 2.20 | 0.03 | 0.05 | -4.18 |
| 15 | PRELP | 0.29 | -0.00 | 2.06 | 0.04 | 0.07 | -4.44 |
| 16 | NTF3 | 0.16 | 0.00 | 1.93 | 0.06 | 0.08 | -4.70 |
| 17 | F3 | -0.15 | -0.00 | -1.92 | 0.06 | 0.08 | -4.71 |
| 18 | LILRA5 | -0.13 | 0.00 | -1.83 | 0.07 | 0.09 | -4.86 |
| 19 | CTSH | -0.09 | 0.00 | -1.50 | 0.14 | 0.17 | -5.38 |
| 20 | ADGRG2 | -0.13 | 0.00 | -1.45 | 0.15 | 0.18 | -5.46 |
| 21 | EGFR | -0.23 | -0.00 | -1.36 | 0.18 | 0.20 | -5.59 |
| 22 | CPA1 | -0.06 | -0.00 | -0.97 | 0.34 | 0.35 | -6.03 |
| 23 | TFRC | -0.06 | 0.00 | -0.84 | 0.41 | 0.41 | -6.14 |

**Table 5**: Differential expression analysis for afternoon sleep TST between 12:00 and 19:00.

| Unnamed: 0 | Assay | logFC | AveExpr | t | P | Adjusted P | B |
|---|---|---|---|---|---|---|---|
| 1 | **IL5** | 0.25 | 0.00 | 4.64 | 0.00 | 0.00 | 2.55 |
| 2 | **CXCL8** | 0.27 | -0.00 | 3.43 | 0.00 | 0.00 | -1.19 |
| 3 | **TGFB1** | 0.37 | 0.00 | 3.28 | 0.00 | 0.00 | -1.61 |
| 4 | **CDCP1** | 0.26 | -0.00 | 2.87 | 0.01 | 0.01 | -2.70 |
| 5 | **CCL3** | 0.09 | 0.79 | 2.14 | 0.04 | 0.04 | -4.33 |
